# Analysis of co-authorship networks among Brazilian graduate programs in computer science

**DOI:** 10.1371/journal.pone.0261200

**Published:** 2022-01-18

**Authors:** Alex Nunes da Silva, Matheus Montanini Breve, Jesús Pascual Mena-Chalco, Fabrício Martins Lopes

**Affiliations:** 1 Computer Science Department, Universidade Tecnológica Federal do Paraná (UTFPR), Cornélio Procópio, PR, Brazil; 2 Center for Mathematics, Computing, and Cognition, Universidade Federal do ABC (UFABC), Santo André, SP, Brazil; University of Sao Paulo, BRAZIL

## Abstract

The growth and popularization of platforms on scientific production has been the subject of several studies, producing relevant analyses of co-authorship behavior among groups of researchers. Researchers and their scientific productions can be analysed as co-authorship social networks, so researchers are linked through common publications. In this context, co-authoring networks can be analysed to find patterns that can describe or characterize them. This work presents the analysis and characterization of co-authorship networks of academic Brazilian graduate programs in computer science. Data from Brazilian researchers were collected and modeled as co-authoring networks among the graduate programs that researchers take part in. Each network topology was analysed with complex network measurements and three proposed qualitative indices that evaluate the publication’s quality. In addition, the co-authorship networks of the computer science graduate programs were characterized in relation to the assessment received by CAPES, which attributes a qualitative grade to the graduate programs in Brazil. The results show the most relevant topological measurements for the program’s characterization and the evaluations received by the programs in different qualitative degrees, relating the main topological patterns of the co-authorship networks and the CAPES grades of the Brazilian graduate programs in computer science.

## Introduction

Social networks have attracted a great deal of attention for decades. Some studies on this topic date as far back as the early 30s and were then mostly done by anthropologists and sociologists [1, 2]. With the increasing use of graph theory to represent social constructs [3], the concepts of small-world [4] and scale-free [5] networks, complex networks [6, 7], and their applications in different contexts, social networks have now drawn the attention of researchers from diverse disciplines, such as computer science, biology, mathematics, chemistry and physics.

One aspect of this research, namely the parallels between social networks and academic collaborations has not gone unnoticed. Research collaboration can be carried out at different levels by researchers with some common goals to co-produce new scientific knowledge [8]. The limits of research collaboration may still be somewhat diffuse given the different forms of interaction between research actors. In this context, the tangible results of collaborations, such as scientific publications, have become important elements to study and analyse collaborations. Academic social networks are heterogeneous networks composed of entities that represent academic actors (e.g., researcher, institution, research group) or products of the result of the performance of these actors (e.g., conference paper, journal paper, book). The analysis of academic social networks allow to observe and study the way of communication and interaction between academic entities, as well as the dissemination of scientific knowledge [9].

A significant number of analyses on academic collaborations between researchers have been made since the establishment of the field of “Scientometrics” in the 70s, although works with similar ideas date as further back as the early 20th century [10]. Some examples of analyses of academic collaborations include nation-wide investigations, such as in Slovenia [11], Brazil [12–16], Germany [17] and Turkey [18]. Other works restrict the analysis to a certain discipline within a country, for example, conducting an analysis of only computer science publications in Brazil [19]. Limiting the scope to certain databases is also common, with, for example, the Zentralblatt MATH database in Germany being explored in [20] or two of Newman’s works [21, 22], where publications between 1995 and 1999 in the areas of physics, biomedical research, and computer science in four specific databases were studied. International collaborations have also been researched, as in [23–25].

There is a rising competitiveness within academia [26], which leads to the development of indicators and world university rankings—such as the THE, QS and Shanghai rankings—or in the many ways to rank researchers based on their academic production with citation and productivity metrics, such as the SCI, *h*-index and PlumX. These metrics are now used worldwide for important decisions concerning funding, hiring and research directions in academia, a path that has led to criticisms [27, 28].

The majority of the contributions in this field, however, address the productivity of individual researchers, oftentimes analyzing how their academic collaboration networks evolve with time. For example, in [29] the question of whether more collaborative researchers tend to have more scientific impact was answered by analyzing their collaboration networks, with [30, 31] and [32] addressing similar questions. Few contributions focus on the performance of specific institutions or groups, such as universities or graduate programs. For example, high-quality Brazilian graduate programs were compared to international programs of excellence based on different universities and citations rankings in [33]. The relationship between a governmental quality assessment and internal academic collaborations among researchers in Brazilian computer science graduate programs was analysed with data from the DBLP database in [34]. Both [34, 33] use the results of the evaluation done by the governmental institution CAPES as a basis for comparison.

The evaluation of Brazilian graduate programs is performed by the Coordination of Superior Level Staff Improvement (CAPES), a governmental institution of the Ministry of Education. The evaluation process takes into account several aspects, such as academic personnel, ongoing research projects, program curriculum, academic production, regional economic and social impacts. The results are released every 4 years and each graduate program is granted a score, called CAPES grade. These vary between 1 and 7 with the latter being the highest possible grade. Since all graduate programs must be evaluated by CAPES and their grades determine whether they can continue to operate and, to an extent, how much government funding they receive, it would be interesting to investigate how the structure of academic collaboration within graduate programs correlate to their CAPES grade.

In this context, this work presents an approach for the analysis of co-authorship networks of Brazilian graduate programs in Computer Science. Thus, the aim of this work is to characterize and to identify topological patterns that correlate with the grade received from CAPES, considering three evaluation periods. For this, complex network and vulnerability measurements were adopted. The co-authorship networks were built based on collected data from the Lattes [35] and Sucupira platforms [36], which, respectively, contain the publications by Brazilian researchers and in which graduate programs they are currently active.

The achieved results can be of great relevance for the coordination of graduate programs, given the factors that differentiate the best evaluated from the least evaluated programs. Thus, being able to improve the understanding of the evaluations received by graduate programs and suggesting the topological patterns in the co-authorship networks that can help the coordinators of graduate programs visualize possible improvements in their programs and work towards better CAPES grades, i.e., with direct propositional actions in their respective programs.

## Materials and methods

**Fig 1:**
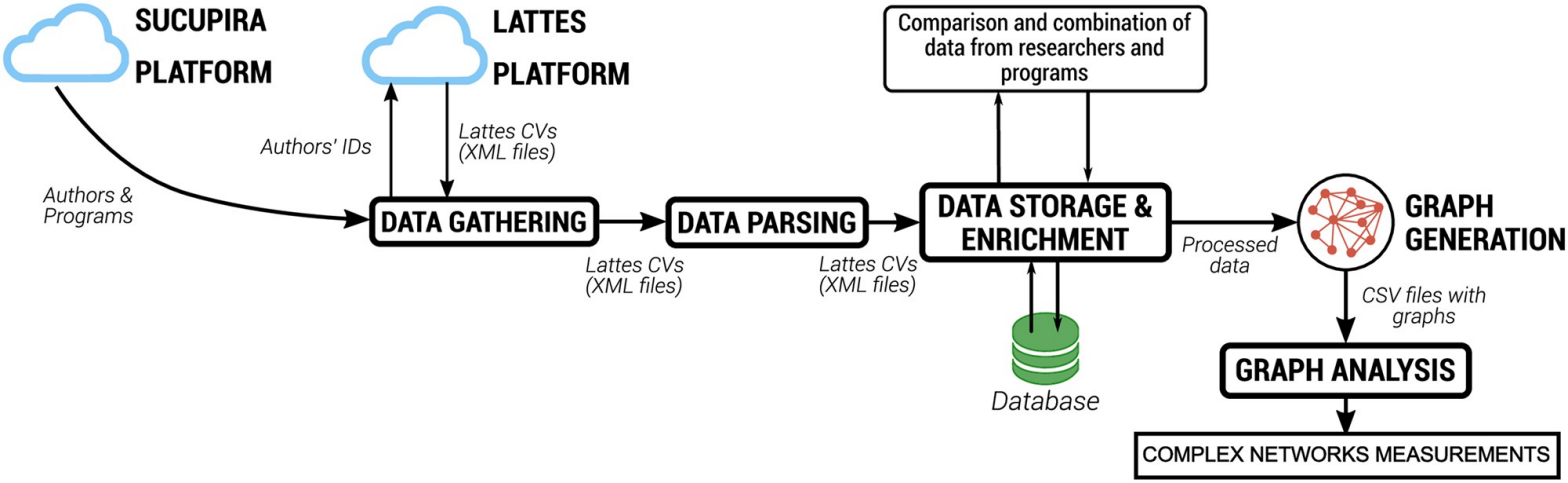
Process flow. Clouds show internet data access. Blocks in bold represent processes. Artifacts generated by each process are described next to the arrows that show the directions of the flow.

This section presents the materials and methods adopted to extract and process data in order to generate the academic co-authorship networks. These networks are then analyzed using complex network measurements. Fig 1 presents an overview of the proposed approach.

### Data sources

The Lattes [35] is a Brazilian online platform where researchers can create their academic resumes and list their publications, research projects, etc. This platform is used throughout the country as a decision factor for hiring university staff, distributing federal financial support for research, and university scoring, among others. Researchers are also evaluated based on their production listed in Lattes resumes, a determining factor for obtaining research grants, for example. As a result, government institutions encourage researchers to keep their resumes up to date and complete. The Lattes platform has over 6 million online resumes. It provides a significant amount of reliable data, which can be extracted and analyzed to determine the key features that distinguish low and high-ranking universities based on the academic performance of their researchers.

The Lattes Resume of 1,644 researchers, affiliated with Brazilian graduate programs in Computer Science, were extracted from the Lattes Platform. The data acquisition process is further detailed in Sec. Data gathering.

The quality of Brazilian graduate programs is assessed since 1998 by CAPES and the results of the evaluation are publicly accessible on the CAPES website. The CAPES assessment is carried out with internal analyses with information systems and professionals who, through studies, reach the CAPES grade [37].

The assessment is carried out in periods, and until 2012 the periods were 3 years; however, due to CAPES changes, from 2013 the assessment period is every 4 years. The CAPES evaluation is carried out by specialized committees in each area of knowledge. Currently, CAPES has 49 evaluation areas [38], which publish specific documents with the guiding criteria for evaluation following common guidelines, however also presents their particularities in the evaluation of each area. In addition, considering the CAPES grades, each graduate program is evaluated relatively within each area of knowledge, i.e. the programs of the same area are compared among themselves for the attribution of grades within each area. The grade received leads to several consequences for the graduate programs, such as programs with grades 1 and 2 are not recommended by CAPES, programs with grades 3 may offer only masters courses, programs with grades 4 or higher may offer masters and PhD courses, programs with grades 5 or higher may participate in some government research funding initiatives, while others do not, to name a few. Therefore, for the graduate programs in Brazil it is mandatory the evaluation and the attribution of the CAPES grade. More detailed technical data on how CAPES performs this assessment can be accessed through the area document and the evaluation form [39, 40].

### Data gathering

A significant contribution of this work is the creation of the database, because there is no open site that makes the data available in an integrated way. It was necessary to extract the data from some platforms, which are presented below. As a result, the data was integrated and stored in a single source.

The Lattes, besides data on the professional performance of researchers and their publications, their academic affiliation is not always made clear. To find out about the researchers that belong to an institution and graduate program, 89,255 Brazilian researchers’ records were gathered from the Sucupira [36]. The Sucupira platform is an important platform to collect information, perform analysis and assessments and be the reference base of the National Post-Graduation System (SNPG) in Brazil. The Sucupira platform makes available the information, processes and evaluation procedures that CAPES performs openly. From this platform, the records of 89,255 Brazilian researchers were extracted.

The data from the Sucupira platform allowed creating a list of graduate programs and their respective researchers. This list was filtered to include only researchers in Computer Science and academic-oriented graduate programs, thus leaving out all other researchers from other areas or professional-oriented programs. A list of 1,644 Computer Science researchers’ full names and their affiliations was produced with this process. Using this approach, even if an international collaboration has been carried out, if 2 or more researchers are linked to the programs, it will be considered in our analyses.

The scope of this work is the Brazilian Computer Science programs, however, the method applied in it can be extended and applied to other areas and researchers in further works.

### Data parsing and storage

The data parsing started by obtaining the academic resumes of the researchers from the Lattes platform, which were identified in the list produced in the previous section. More specifically, the resumes were downloaded from Lattes platform in an XML format. The XML data were converted into structured data (DBMS). As a result, a SQL-based database management system was produced.

Regarding the publications available on the Lattes platform, there are several types, such as articles published in journals, full papers published in conference proceedings, abstracts published in conference proceedings, books, book chapters, among others. In this work, publications in journals and full papers in conference proceedings were used to study and analyze research collaboration among researchers associated with Brazilian graduate programs in computer science (both types of publications are the most common tangible output of a research collaboration).

Therefore, the information about the list of researchers, the respective graduate programs to which they are linked, their institution and their intellectual production were stored and reviewed in a database. In this way, CAPES evaluates each of the programs and assigns them a grade for each time interval. This information was entered and indexed to the graduate programs for further analysis. The generated database, as well as the algorithms and filters applied to the data (step by step) are freely available at: https://github.com/alexjrns/datamining_lattes_computer_science.

### Graph generation

One challenge in building a co-authorship network is to extract the data from a source and correctly attribute the publications to the respective authors. Names may contain errors, for example, names written with distinct characters, with abbreviations, without accents, or the existence of homonyms, leading to unreliable relationships [41].

To circumvent this problem, all researchers’ names were normalized (i.e., names were transformed to lowercase, no accents and no punctuation marks). The correct identification of the researchers was carried out through the approximate matching of normalized names using the Levenshtein’s distance [42, 43]. The comparison between the names of the authors was performed to remove or reduce the number of ambiguities, leading to improvement in the quality of collaborative relationships [19, 44]. The adopted approach comprises analyzing two strings A and B and returning the number of operations required to transform string A into a string B. If the number of operations is less than or equal to 2, it is understood that the strings match. Only full names are stored in the Lattes and Sucupira platforms, allowing us to reach a high percentage of perfect matches. In fact, 1529 (93%) of the 1644 researchers were identified directly without any ambiguities. Using the data from Lattes to filter the matched names to include only researchers belonging to the field of computer science allowed us to clear many of the ambiguities and, in the end, only 3% of all researchers were not identified automatically.

The academic collaboration networks were produced considering the researchers as nodes in this network. However, the networks were analyzed considering the respective graduate programs that the researchers are associated with. Thus, the researchers are represented by nodes, and the articles published in collaboration between two researchers represent an edge in the produced graph. Thus, both the collaboration within the graduate program (subgraph formed only by the researchers of each program), and the collaboration between researchers belonging to different programs (collaboration between subgraphs) were considered in this work.

Another relevant aspect is the analysis of the dynamics of each graduate program. This work considers three evaluative periods by CAPES between 2007-2009, 2010-2012 and 2013-2016 and their respective CAPES grades. The evaluation between 2017-2020 is ongoing and not yet available from CAPES. Therefore, the analysis of dynamics helps to identify which properties, in terms of academic collaborations, lead a program to receive the same evaluation or better/worse evaluations in different time windows.

### Graph analysis

Complex network theory has been successfully applied in many areas, particularly within representation of networks of different types, such as biological systems [45–48], computer vision [49–52], the electric power grid [53], the Internet [54], subway systems [55], and neural networks [56], to cite but a few. Another area in which they are also applied is the representation of friendship networks or collaborations between individuals.

The complex network measurements can represent and characterize specific topological models [57]. Therefore, these measurements can be applied in the characterization of topological patterns in the networks. In this context, 42 complex networks measurements [58] were considered in this work, such as Number of Nodes, Number of Edges, Betweenness Centrality, Cluster Coefficient, Average Path Length, among others in order to explore and analyse the relationships of the topological patterns and the CAPES grades. Among the measures considered are measures of complex network structure (topology), analysis of vulnerability measures and measures that assess the position in which the researcher’s name is among the authors of the publication.

In order to analyze the topological patterns of co-authorship networks, a feature matrix is generated by composing the complex networks measurements and the respective CAPES grades. More specifically, the measures were organized as feature vectors with size *n* + 1, where *n* is the total number of features, and CAPES grade (cg) was added in the last column referring to the analyzed program and the observed period of evaluation. Thus, feature vectors were produced for each graduate program and each period of time evaluation and its respective CAPES grade and arranged into a feature matrix *M*_*f*_ as follows:

$$M_{\text{f}}=\left[\begin{array}{cccccc} f_{1,1} & f_{2,1} & f_{3,1} & \ldots & f_{\text{n},1} & cg_{1}\\ f_{1,2} & f_{2,2} & f_{3,2} & \ldots & f_{\text{n},2} & cg_{2}\\ \vdots & \vdots & \vdots & \ddots & \vdots & \vdots \\ f_{1,\text{m}} & f_{2,\text{m}} & f_{3,\text{m}} & \ldots & f_{\text{n},\text{m}} & cg_{\text{m}} \end{array}\right]$$

### Classification

From the feature matrix, classification algorithms applied to identify which features lead to an adequate classification and generalization considering the respective CAPES grade. The framework Weka [59] and the Random Forest (RF) algorithm [60] with default parameters were adopted. RF is a decision tree algorithm and allows a direct interpretation of its results by recovering the rules applied in the classification process. Thus, random forest allows the retrieval of the adopted features by the classifier, which was one goal of this work and the estimating the importance of these features. The 10-fold cross-validation was adopted as the validation method as described in [61]. As a result, it is possible to recover the importance of each feature in the classification of CAPES grade, i.e. it is possible to observe which feature has relevance for the correct classification.

**Fig 2:**
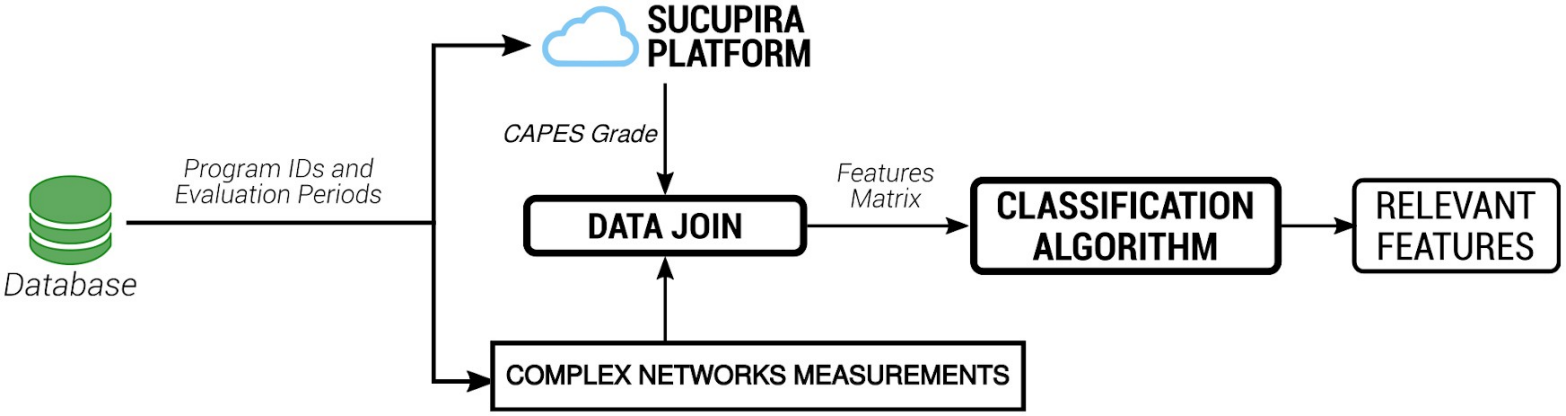
Process flow for topological metrics analysis. Note that the data saved in the database is used as a filter for downloading CAPES grade from the internet and identifies each program with its topological measurements. This data is gathered into a feature matrix and the algorithms performed.

The overview of this process are presented in Fig 2.

### Author order

The order in which authors are listed in a publication can provide information that can be analysed and discussed. This order follows distinct patterns, depending on the research field or country. A straightforward way is the alphabetical sorting of the authors. However, this is not typical behavior in computer science, and in other research areas, in which authors are ordered according to their contribution [62–64]. First author is the one that used a more considerable effort, defined the materials, methods, and objectives of the work and realizes the final analysis of the results. Last author quoted commonly is the supervisor of the work and the project leader. The authors who are not in first or last authorship are co-authors who contributed to the work, but specifically in certain points. The analyses of these co-authorship orders can be used as qualitative measures of a group of authors’ publications.

The analysis on researchers’ performance considering the effects of seniority, their respective genders, and their geographic positions was performed [65]. More specifically, academic publications and citations were analyzed based on the Scopus, Web of Science and European Research Council (ERC) collaboration network, which covered 355 Life Science scholars in the period from 2007 to 2009. The authors considered 2 types of researchers, the first being those who were called juniors who are the researchers who are starting or consolidating their research team. They also considered the so-called senior researchers, who are those with a significant research history in the last 10 years. With the results, it was possible to observe that although all researchers had an increase in the size of the collaboration networks and the number of sub-communities during the time analyzed, the growth in juniors was greater. It was also possible to see that in both groups, the collaboration network was enlarged from the grant award to the researchers.

In order to investigate this context, this work proposes 3 qualitative indexes to evaluate the order of citation of the authors. The indexes are the *First Author Index*, which is the proportion of all the publications in which the authors of the graduate program are the first. The *Collaboration Index* is the proportion of publications in which the researcher is cited at the middle (neither first nor last) and the *Seniority Index* is the proportional of publications in which the researchers are the last author in a publication. The proposal is to compare these indexes with the CAPES grade and analyse its relation with the proposed indexes.

## Results and discussion

The first step was to perform a normalization throughout the feature matrix in order to adjust each feature to the range from 0 to 1. It is an important issue so that classification algorithms did not suffer interference by the range of the feature values.

Another important issue is the unbalanced dataset. The produced dataset contains 171 computer science graduate programs in which 75 programs with CAPES grade 3, 58 programs with grade 4, 14 programs with grade 5, 9 programs with grade 6, and 15 programs with grade 7. Thus, the different number of samples per class (CAPES grade) can influence the classification algorithms. In this context, subsets of 15 graduate programs were created considering the graduate programs with grades 3 and 4 equally distributed. All experiments were performed for each subgroup, and the average of the subsets results was considered. It is important to note that the results refer to the last 3 evaluated periods.

**Fig 3:**
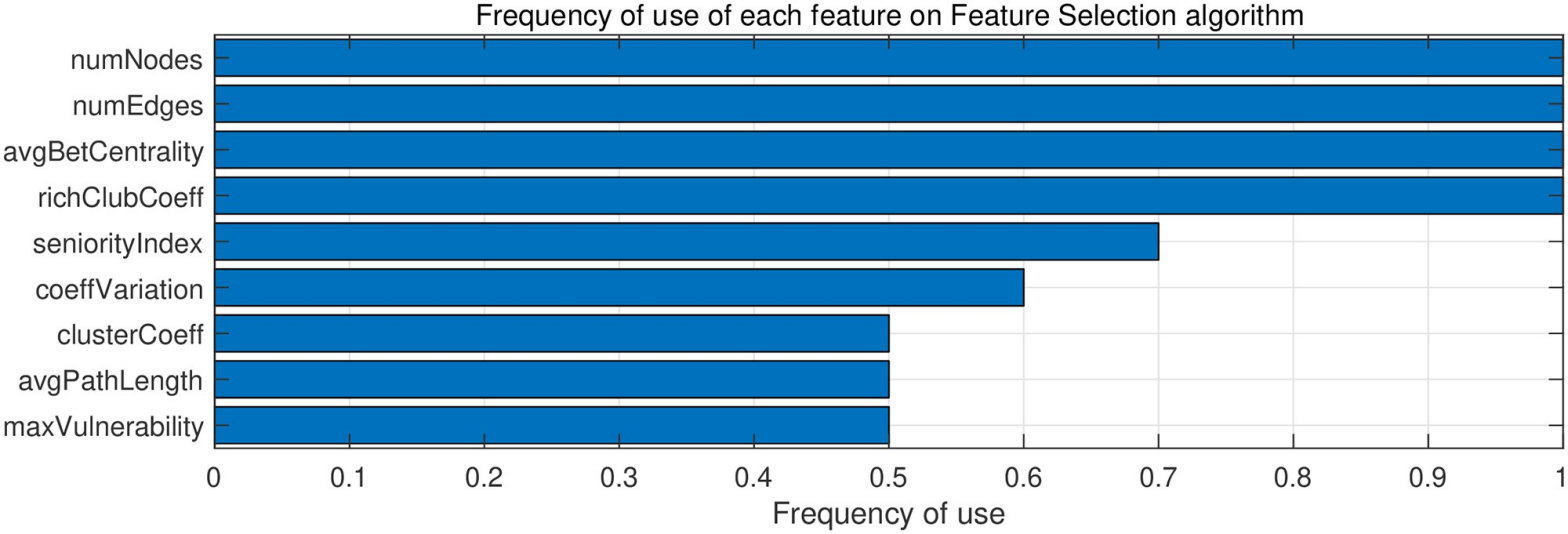
Importance of each feature for the classification of CAPES grade regarding the feature selection algorithm.

Considering the re-scaled complex network measurements, and the balanced subsets of graduate programs, the feature selection algorithm was performed in order to analyse which features better describe the CAPES grade. The CfsSubSetEval feature selection and BestFirst as the search method available at Weka [59] with its default parameters adopted. The CfsSubSetEval [66] is a correlation-based feature selection, which evaluates the value of a subset of attributes by considering the individual predictive ability of each feature, along with redundancy between them. Thus, subsets of features that are highly correlated with the class, yet have low inter-correlation, are preferred. Thus, the results showed that some features have a high association with the CAPES grade. More specifically, 9 features have a significant correlation with the CAPES grade. Fig 3 presents the selected features with the percentage of times each feature was selected. An explanation about the selected features and its properties are discussed in the following.

- *Number of Nodes*: This measure deals with the number of nodes in each network, in this case, the number of researchers in each program. Thus, programs with the highest grade is the programs with the largest number of researchers, so this measure is consistent with the analysis, where the higher the number of nodes (researchers), the greater the CAPES grade;
- *Number of Edges*: This measure refers to the number of connections in the network, i.e. the number of publications among the researchers. Programs with larger CAPES grade are programs that have more works published in collaboration with their researchers. Therefore, the best-evaluated programs have greater internal collaboration compared to the others;
- *Average Betweenness Centrality*: This index deals with the nodes’ centrality in a network because it analyses the nodes in the shortest path between two connected nodes. The higher the average of this measurement, the more researchers are taking part in shorter paths. More specifically, the betweenness centrality quantifies the relevance of a researcher in relation to co-authorships of the network, i.e. the more publications an author has in collaboration, the greater will be your betweenness. Thus, an author with a higher betweenness centrality represents the greater participation in the publications of the program. As the aim of our work was to evaluate the graduate programs regarding the CAPES grade, it was adopted the average betweenness centrality [57] of each program;
- *Rich Club Coefficient*: This index measures the proportion in which the nodes with a significant number of connections (hubs) of a network are connected. This coefficient can evaluate the robustness of a network, because the higher the value, the more strongly connected, which shows that if one of these hubs is removed, the lower the impact on the network structure. In this work, this measure evaluates the tolerance to changes of a program if a researcher is randomly removed, programs with lower CAPES grade have a greater dependence on their researchers, in case a vital researcher is removed from the program, the structure of the program will be strongly affected, different from the best-evaluated programs, that have less individual dependence of researchers;
- *Seniority Index*: This measure is proposed in order to evaluate the percentage of publications in which the researcher has the last name in a publication. Thus, it is possible to qualify the publications of each researcher and therefore generalize this measure as the average of the Seniority Index of all researchers in the same graduate program. It is possible to notice that higher average seniority index refers to the graduate programs with higher CAPES grade.
- *Variation Coefficient*: The Coefficient of Variation measures the variation of a network; its mathematical equation results from the standard deviation of the values of the network divided by the average of these values. Regarding this work, this metric informs how much the adopted measures vary in each one of the graduate programs;
- *Cluster Coefficient*: This index shows a tendency in which the graph nodes have to group and form subsets. In social networks, these clusters are communities of individuals that share common features. In this work, a cluster is a group of researchers that have research projects in common, so the higher the value of this metric, the greater the number of publications among the researchers of this program (internal collaboration). Programs with higher CAPES grade have greater internal collaboration (more edges);
- *Average Path Length*: This measure performs the average path of the network as the average number of steps in the shortest paths for all nodes’ pairs. The lower this indicator, the higher the efficiency in transporting information inside the network. In this work, this measure represents the average number of authors connecting an Author X to an Author Y, assuming that both do not have a direct connection, so the smaller the measure, the easier it is to connect 2 directly disconnected researchers. The results show that programs with lower evaluations of CAPES grade have a lower value in this measure. Better-evaluated programs have a higher complexity in the connection of their nodes. Since the number of nodes and the number of edges in better-evaluated programs are higher, there are more paths (co-authorship) and more nodes (researchers) in these networks. However, when this metric is considered relatively, dividing the value of the Average Path of the Network by the number of edges, it is possible to notice that this value is inverted. Therefore, it shows that although the networks have more researchers, they still efficient;
- *Swan Connectivity*: The Swan Connectivity is a measure of network vulnerability [67], which calculates the loss of connectivity when a vertex is removed from the network. As a result, measures the decrease in the number of relationships between each vertex of the network when one vertex or several are removed. In this work, this measure shows how vulnerable a network can be because when removing a researcher (vertex), the network loses connectivity, which is a particularity of graduate programs with lower CAPES grade.

The second experiment was performed in order to analyse the importance of features into the classification process. Weka [59] and its Random Forest classification algorithm with default parameters was performed by adopting the 10-fold cross-validation. A suitable way to assess the performance of classifiers algorithms is the area under the receiver operating characteristic (ROC) curve (AUC) [68]. Table 1 shows the classification results. It is possible to notice that the AUC was superior to 0.7 for all CAPES grade, achieving 0.828 for CAPES grade 3 and 0.929 for CAPES grade 7.

**Table 1:** Area under the receiver operating characteristic (ROC) curve (AUC) for the Random Forest applied on feature matrix.

CAPES grade	AUC
3	0.828
4	0.701
5	0.741
6	0.838
7	0.929
**Average**	**0.787**

**Fig 4:**
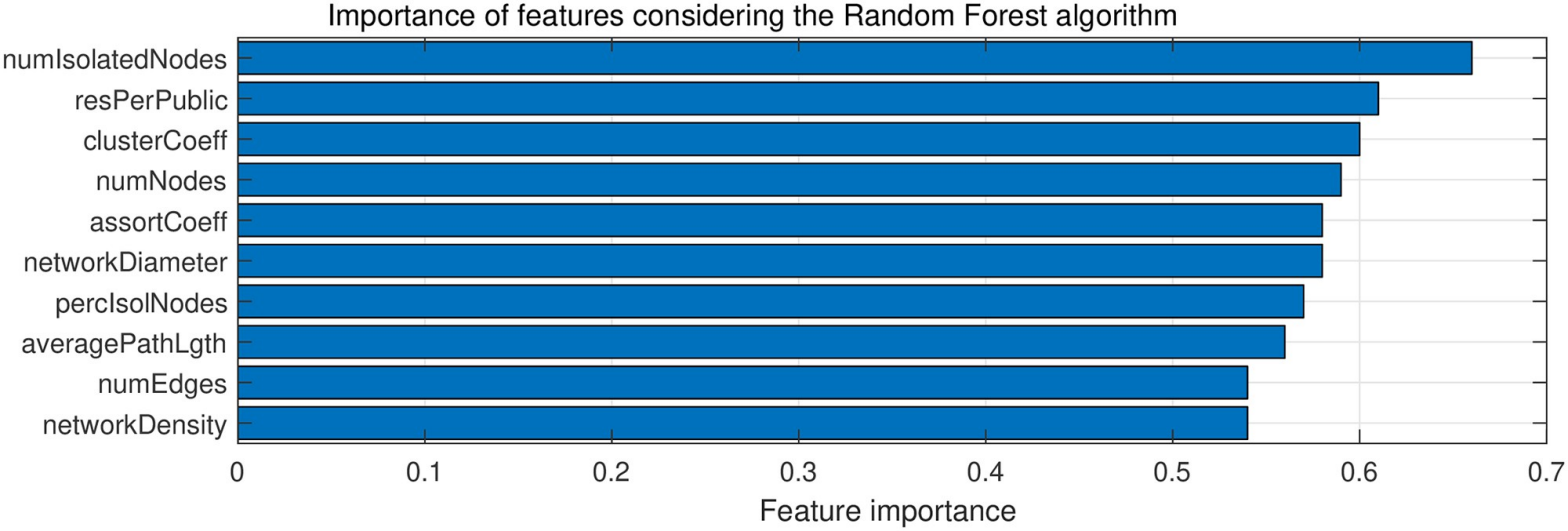
Importance of each feature for the classification of CAPES grade regarding the Random Forest algorithm.

Fig 4 shows the percentage of times each feature was selected. It is possible to observe that the most relevant feature in this context is the number of isolated nodes, researchers that have no work in common with their colleagues in the graduate program, which was a particularity of the graduate programs with higher CAPES grade. The second important feature was researchers per publication, showing that is an important pattern that distinguish the graduate programs. The cluster coefficient, number of nodes, number of edges and average path length reinforce the importance of these features for the identification of CAPES grades.

**Fig 5:**
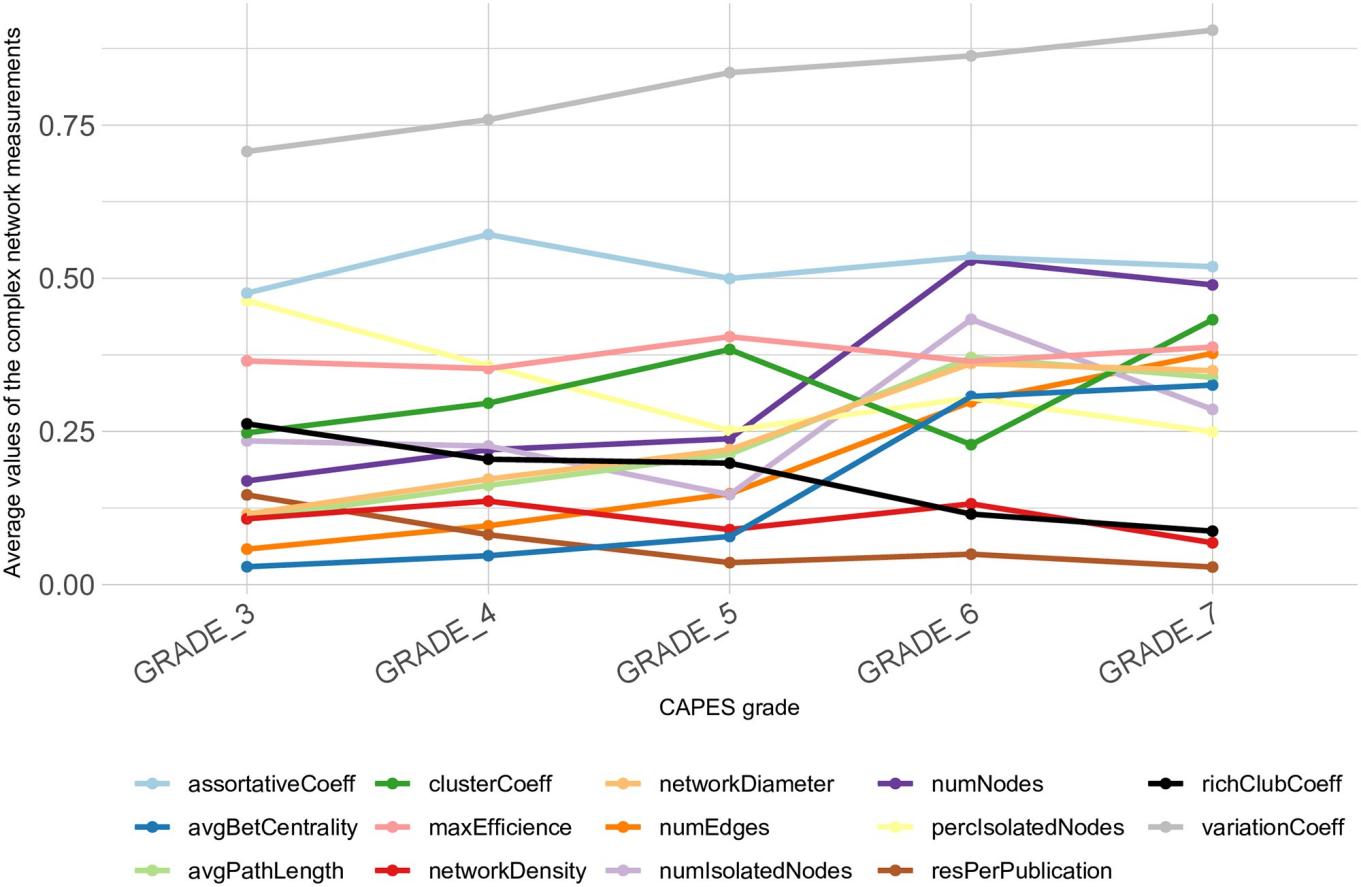
Average of the most important complex network measurements regarding the CAPES grades.

Considering that graduate programs in Brazil evaluated with a CAPES grade of 3 or higher are recommended, which leads to five classes: 3, 4, 5, 6 and 7. In order to better investigate the behavior of the measures identified as important for the characterization of CAPES grades, Fig 5 shows the average of these measures considering the graduate programs classified by the same CAPES grade. It can be seen that all complex networks measurements have variations between CAPES grades. As expected, the number of researchers (numNodes), the number of isolated researchers (numIsolatedNodes) and the number of publications (numEdges) are important measures. However, it can be highlighted the average betweenness centrality (avgBetCentrality) which presents lower values for programs with CAPES grades 3, 4 and 5 and a significant increase in its value occurs for programs with CAPES grades 6 and 7. With similar behavior, but with less intensity, also stand out the average path length (avgPathLength) and network diameter (networkDiameter), which reinforces the increasing distance between researchers in programs with higher CAPES grades. The measure of rich club coefficient (richClubCoeff) can also be highlighted with decreasing behavior as the CAPES grades increase, showing that programs with higher CAPES grades have less individual dependence of researchers. On the other hand, the coefficient of variation (variationCoeff) shows a clear increasing behavior with the increase of CAPES grades. This measurement quantifies the heterogeneity of the vertices in the network, so the more different the vertices are, such as their degree and other measurements, the greater the variation, indicating that better evaluated graduate programs have a greater diversity of researcher profiles.

**Fig 6:**
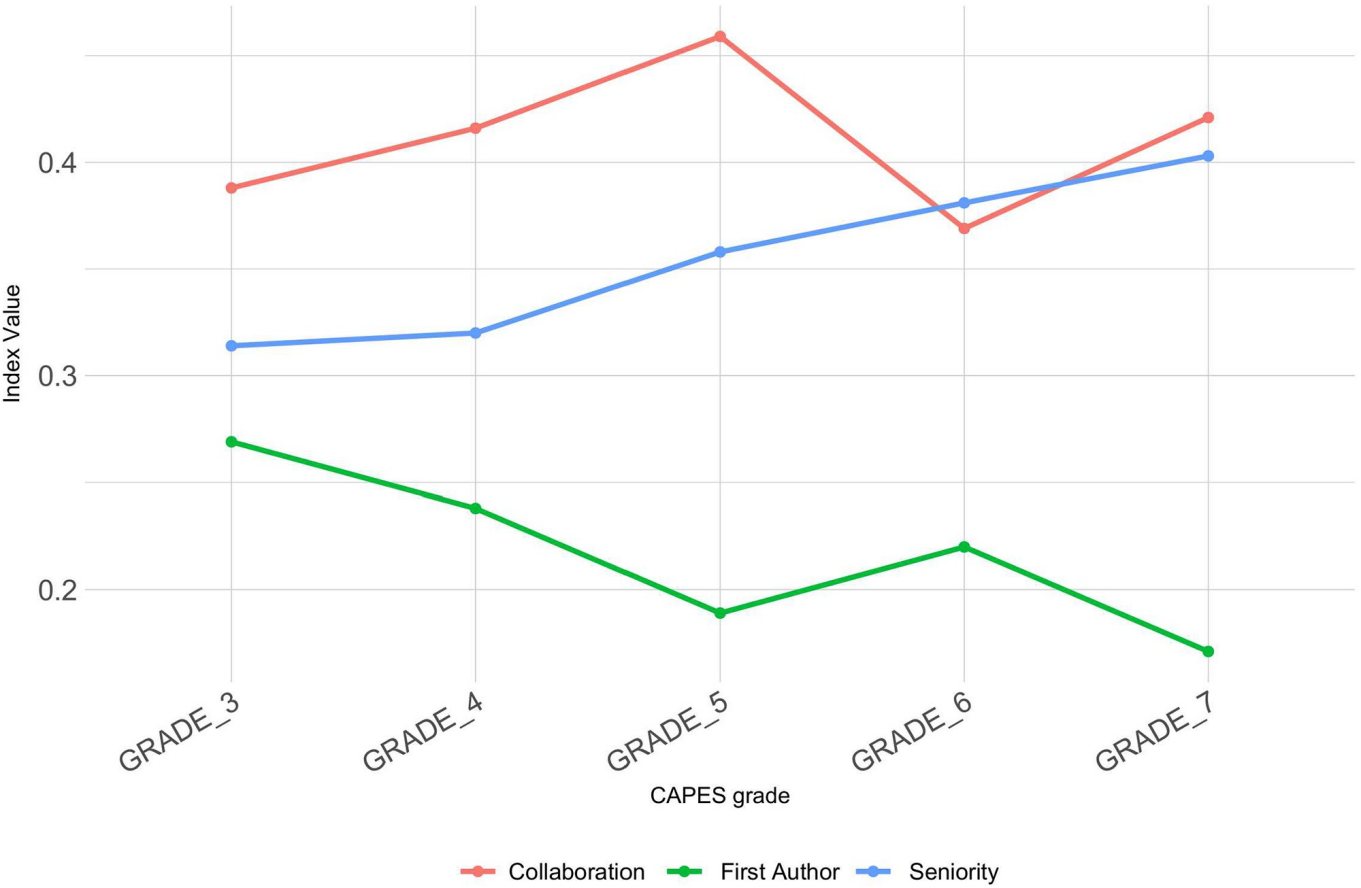
Average of the proposed indexes in graduate programs regarding their CAPES grades.

The similar analysis was also performed by the proposed indexes, thus the average values of each CAPES grade were performed for each proposed index. Fig 6 shows the average values for the 3 proposed indexes considering the CAPES grades. It is possible to observe that the seniority was pointed out by the feature selection algorithm, present a monotonically increasing behavior, i.e. as the CAPES grade of the graduate programs increases, the index also increases. The first author index presents the inverse behavior, with higher value for graduate programs with grade 3 and lower value for graduate programs with grade 7, indicating a pattern of research composition for the graduate programs. Regarding the collaboration index, it is possible to notice that there is an increasing pattern among programs 3, 4 and 5, however a decrease of this index for programs with grades 6 and 7.

**Fig 7:**
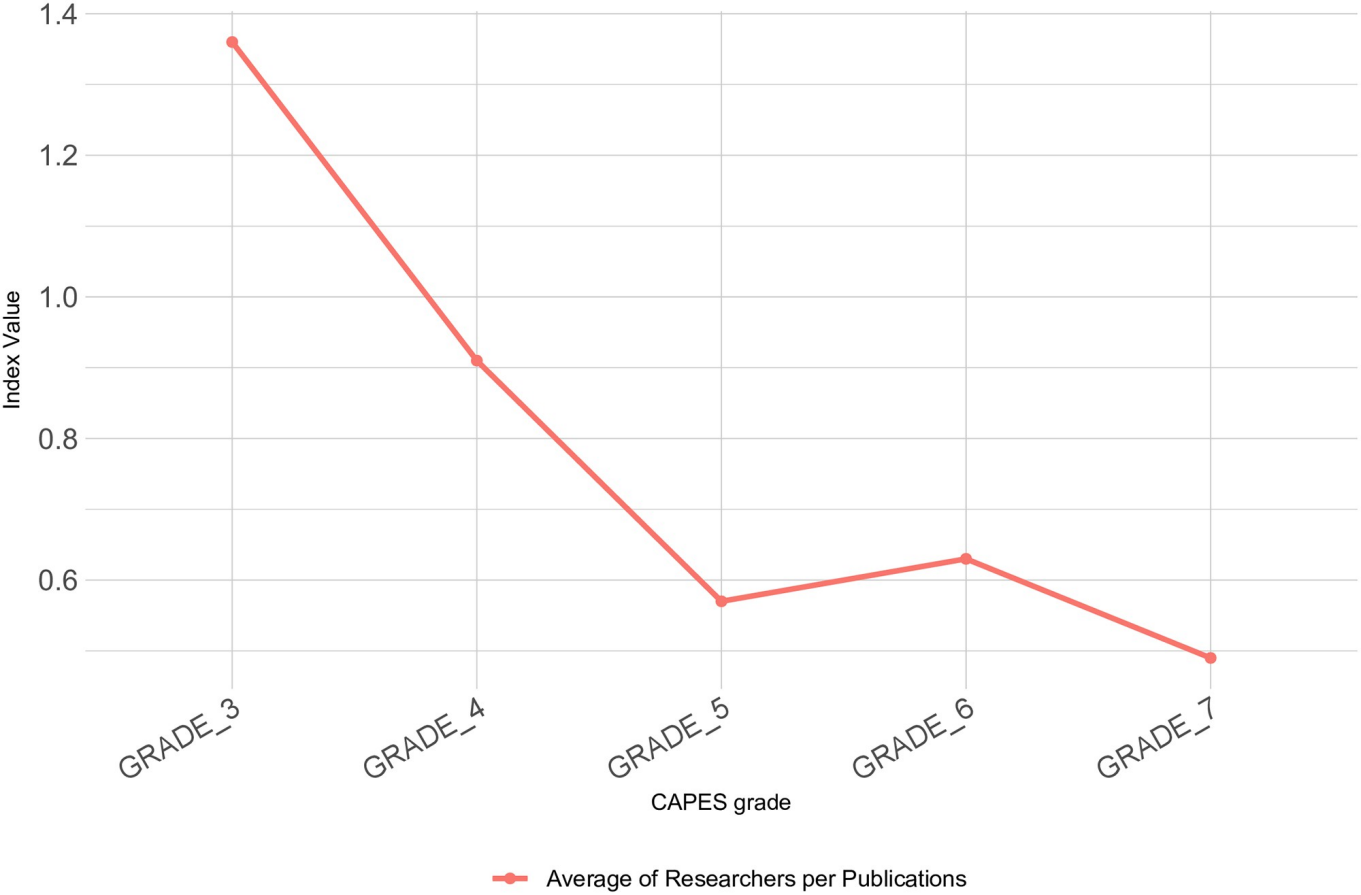
Average number of researchers per publication in graduate programs regarding their CAPES grades.

The random forest algorithm pointed the number of isolated nodes out as an important feature. In order to better investigate this feature, the average values of researchers per publication were adopted. The average values of the graduate programs for each CAPES grade are shown in Fig 7. It can be seen that there is a clear variation in this index between the programs, in which graduate programs with higher CAPES grade have a lower average number of researchers per publication. Thus, it can be noticed that programs with higher CAPES grades are more efficient than the programs with lower CAPES grades, regarding the platforms Lattes and Sucupira provide identification and publications from the Brazilian researchers and graduate programs. Therefore, international researchers are not part of the co-authorship networks adopted in this study.

## Conclusions

This work proposes a complex network approach to analyse and characterize the co-authorships of Brazilian computer science graduate programs. Considering that the Brazilian graduate programs are evaluated relatively by CAPES within each area of knowledge for the attribution of the grade, the computer science graduate program co-authorship networks were analysed, identifying the most relevant network measurements to characterize these graduate programs regarding their quality by considering the CAPES grade. It was analysed 62 Brazilian graduate programs in computer science, with about 1,644 researchers observed in three CAPES evaluation periods from 2007 to 2016. A dataset was produced after the pre-processing the data from Lattes platform. Thus, the produced dataset allows the analyses of graduate programs by considering 42 complex networks measurements regarding their CAPES grades.

The adopted measurements that considering the size of the networks (graduate programs) were the most significant. Thus, the larger the program, either in the number of researchers and in the number of publications, higher are the CAPES grades. However, the higher number of researchers per program must be combined with more publication. Thus, the aim of this work is not only to point out the important features but also to explain how these features can act together in order to explain the CAPES grade.

The feature selection algorithm pointed the measures of centrality (importance) in the networks out. It was observed that better-evaluated graduate programs have more elements with higher centrality, so these programs have more researchers of greater influence. Vulnerability (Rich Club Coefficient and SWAN Connectivity) measures also yielded relevant results, with better-rated programs being less vulnerable than lower-rated ones, i.e. when a researcher is randomly removed, the program structure undergoes fewer changes, which is not the case with lower-rated programs that are more highly rated vulnerable.

The random forest classifier algorithm pointed the number of isolated nodes out as an important feature. Thus, it was possible to observe that graduate programs with higher CAPES grades are more productive because they have more publications per researcher, therefore, more efficient.

Three qualitative measures of collaborative evaluation among the researchers are proposed based on the author order of co-authorship regarding the publications: first, middle or last. The results for such analysis leads to interesting patterns, which graduate programs with lower CAPES grade have a higher first author index than the others. Programs with intermediate grades have a higher collaboration index than others, while the highest-rated programs have a higher seniority index than the others, with a monotonically increasing behavior as the programs evaluation increases.

In summary, this work points out some important patterns to be analysed that lead to the characterization of the graduate programs related to CAPES grade can bring information for the Brazilian computer science community to analyse and to adopt strategies that can lead to the improvement of these patterns and, improve the assessment of the graduate programs. Investigations concerning how the CAPES grade is related to other qualitative indexes available in the literature are needed and suggested as further work.

It is important to highlight that the measures raised in this work reveal the reality of these networks in which the scope of work. When evaluating other networks such as international collaborations or interaction between other programs, these measures may present different values, as in these cases they will be other scenarios and therefore future work can be carried out using the same approach as this one.
