# Correction: Analysis of co-authorship networks among Brazilian graduate programs in computer science

**DOI:** 10.1371/journal.pone.0271937

**Published:** 2022-07-18

**Authors:** The PLoS ONE Staff

The first author’s name is spelled incorrectly. The correct name is: Alex Junior Nunes da Silva. The correct citation is: Nunes da Silva AJ, Breve MM, Mena-Chalco JP, Lopes FM (2022) Analysis of co-authorship networks among Brazilian graduate programs in computer science. PLOS ONE 17(1): e0261200. https://doi.org/10.1371/journal.pone.0261200

The publisher apologizes for the error.
